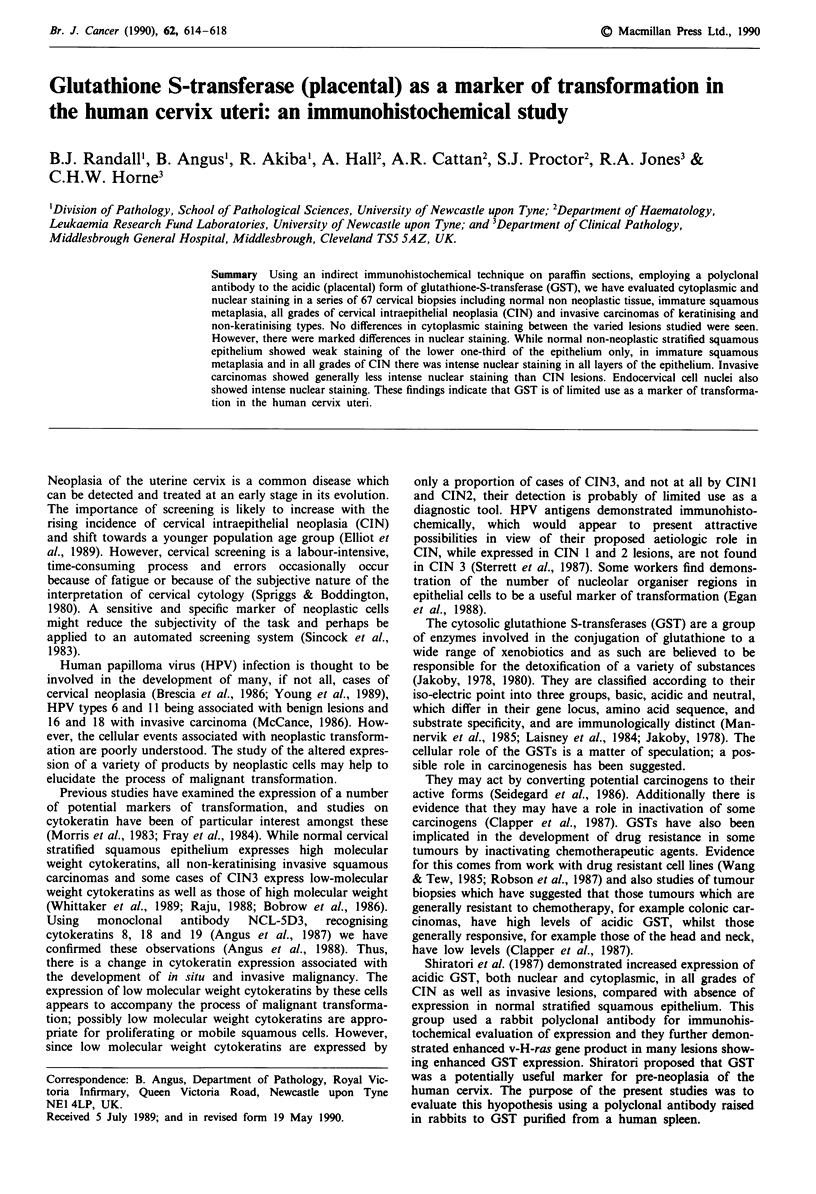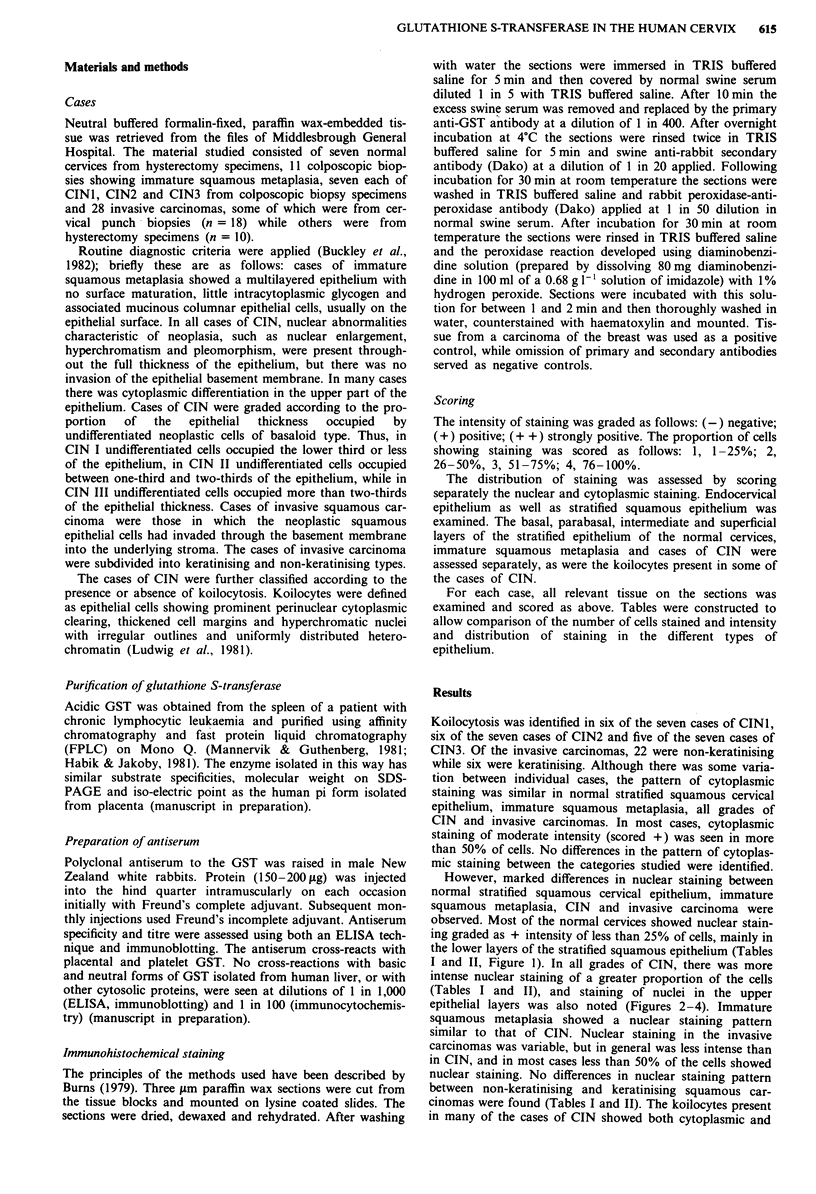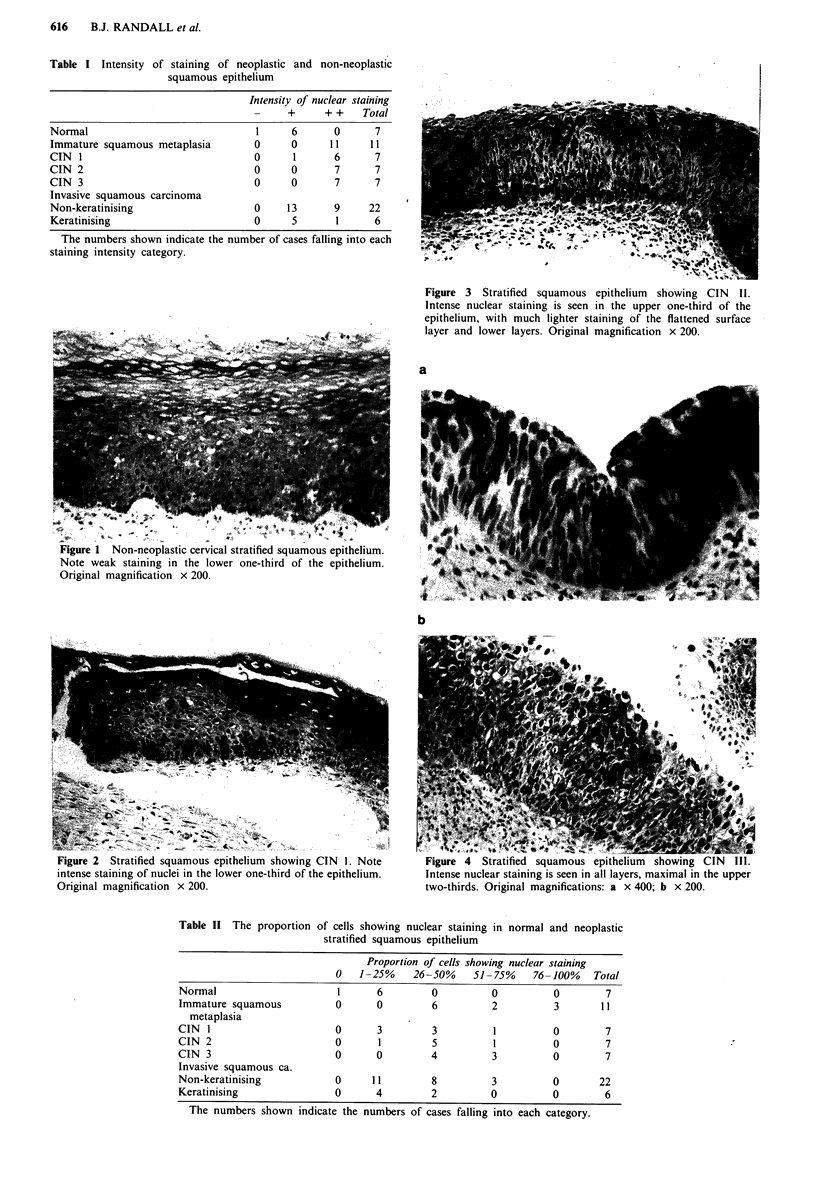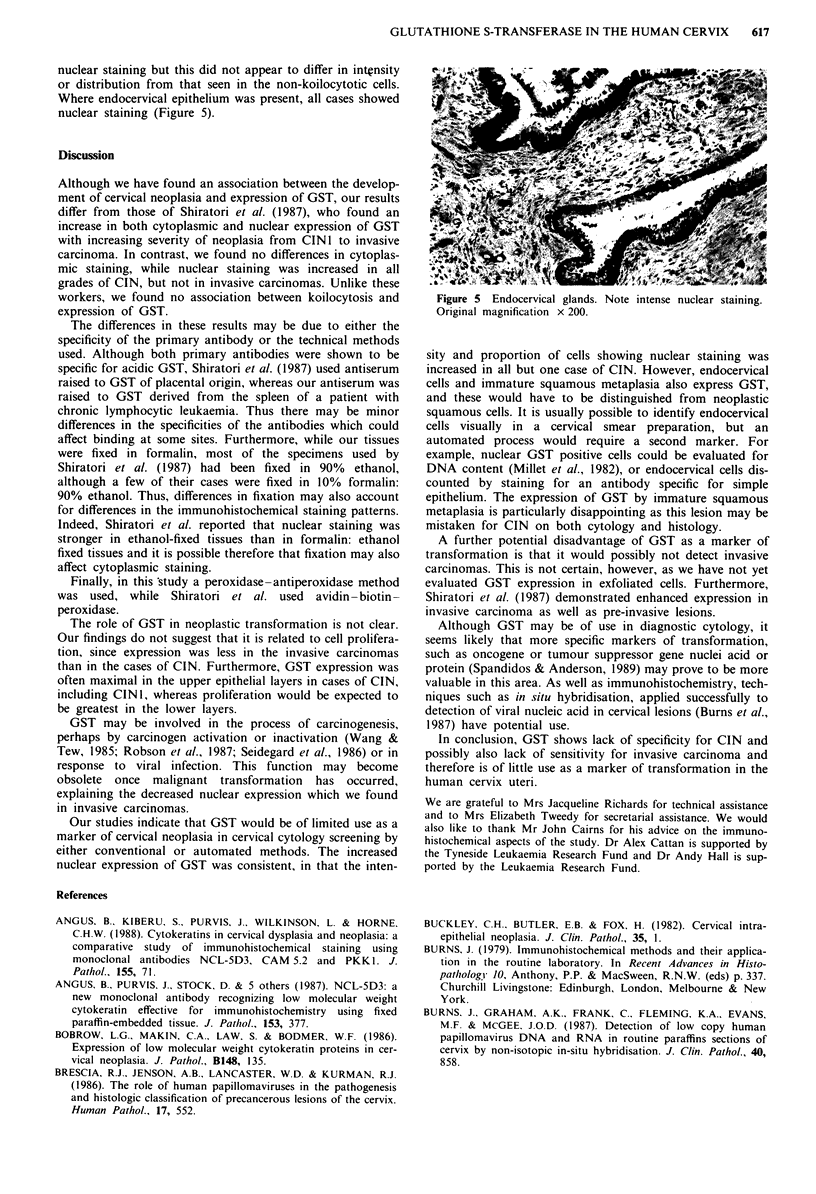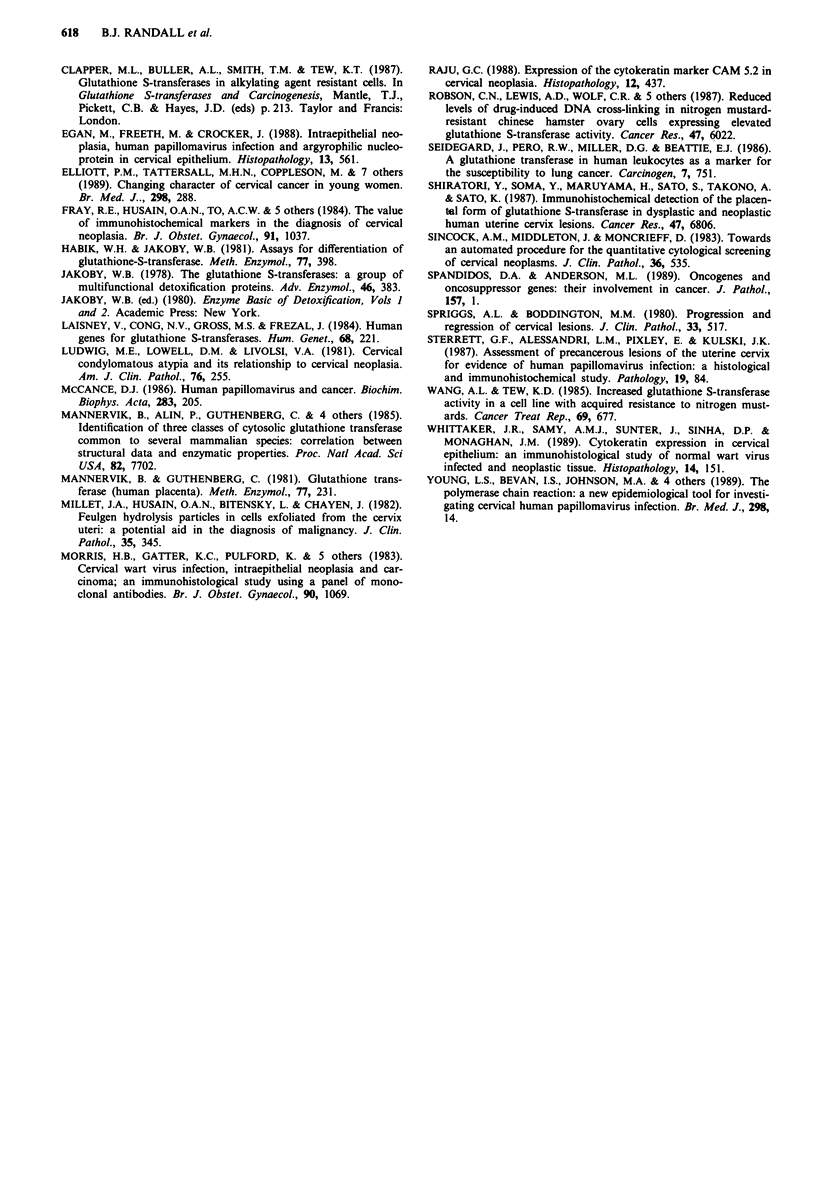# Glutathione S-transferase (placental) as a marker of transformation in the human cervix uteri: an immunohistochemical study.

**DOI:** 10.1038/bjc.1990.340

**Published:** 1990-10

**Authors:** B. J. Randall, B. Angus, R. Akiba, A. Hall, A. R. Cattan, S. J. Proctor, R. A. Jones, C. H. Horne

**Affiliations:** Division of Pathology, School of Pathological Sciences, University of Newcastle upon Tyne, UK.

## Abstract

**Images:**


Br. J. Cancer (1990), 62, 614-618 © Macmillan Press Ltd., 1990

### Glutathione S-transferase (placental) as a marker of transformation in the human cervix uteri: an immunohistochemical study

B.J. Randall[1], B. Angus[1], R. Akiba[1], A. Hall[2], A.R. Cattan[2], S.J. Proctor[2], R.A. Jones[3] & C.H.W. Horne[3]

[1]*Division of Pathology, School of Pathological Sciences, University of Newcastle upon Tyne;* [2]*Department of Haematology, Leukaemia Research Fund Laboratories, University of Newcastle upon Tyne; and* [3]*Department of Clinical Pathology, Middlesbrough General Hospital, Middlesbrough, Cleveland TS5 5AZ, UK.*

**Summary** Using an indirect immunohistochemical technique on paraffin sections, employing a polyclonal antibody to the acidic (placental) form of glutathione-S-transferase (GST), we have evaluated cytoplasmic and nuclear staining in a series of 67 cervical biopsies including normal non neoplastic tissue, immature squamous metaplasia, all grades of cervical intraepithelial neoplasia (CIN) and invasive carcinomas of keratinising and non-keratinising types. No differences in cytoplasmic staining between the varied lesions studied were seen. However, there were marked differences in nuclear staining. While normal non-neoplastic stratified squamous epithelium showed weak staining of the lower one-third of the epithelium only, in immature squamous metaplasia and in all grades of CIN there was intense nuclear staining in all layers of the epithelium. Invasive carcinomas showed generally less intense nuclear staining than CIN lesions. Endocervical cell nuclei also showed intense nuclear staining. These findings indicate that GST is of limited use as a marker of transformation in the human cervix uteri.

Neoplasia of the uterine cervix is a common disease which can be detected and treated at an early stage in its evolution. The importance of screening is likely to increase with the rising incidence of cervical intraepithelial neoplasia (CIN) and shift towards a younger population age group (Elliot *et al.*, 1989). However, cervical screening is a labour-intensive, time-consuming process and errors occasionally occur because of fatigue or because of the subjective nature of the interpretation of cervical cytology (Spriggs & Boddington, 1980). A sensitive and specific marker of neoplastic cells might reduce the subjectivity of the task and perhaps be applied to an automated screening system (Sincock *et al.*, 1983).

Human papilloma virus (HPV) infection is thought to be involved in the development of many, if not all, cases of cervical neoplasia (Brescia *et al.*, 1986; Young *et al.*, 1989), HPV types 6 and 11 being associated with benign lesions and 16 and 18 with invasive carcinoma (McCance, 1986). However, the cellular events associated with neoplastic transformation are poorly understood. The study of the altered expression of a variety of products by neoplastic cells may help to elucidate the process of malignant transformation.

Previous studies have examined the expression of a number of potential markers of transformation, and studies on cytokeratin have been of particular interest amongst these (Morris *et al.*, 1983; Fray *et al.*, 1984). While normal cervical stratified squamous epithelium expresses high molecular weight cytokeratins, all non-keratinising invasive squamous carcinomas and some cases of CIN3 express low-molecular weight cytokeratins as well as those of high molecular weight (Whittaker *et al.*, 1989; Raju, 1988; Bobrow *et al.*, 1986). Using monoclonal antibody NCL-5D3, recognising cytokeratins 8, 18 and 19 (Angus *et al.*, 1987) we have confirmed these observations (Angus *et al.*, 1988). Thus, there is a change in cytokeratin expression associated with the development of *in situ* and invasive malignancy. The expression of low molecular weight cytokeratins by these cells appears to accompany the process of malignant transformation; possibly low molecular weight cytokeratins are appropriate for proliferating or mobile squamous cells. However, since low molecular weight cytokeratins are expressed by only a proportion of cases of CIN3, and not at all by CIN1 and CIN2, their detection is probably of limited use as a diagnostic tool. HPV antigens demonstrated immunohistochemically, which would appear to present attractive possibilities in view of their proposed aetiologic role in CIN, while expressed in CIN 1 and 2 lesions, are not found in CIN 3 (Sterrett *et al.*, 1987). Some workers find demonstration of the number of nucleolar organiser regions in epithelial cells to be a useful marker of transformation (Egan *et al.*, 1988).

The cytosolic glutathione S-transferases (GST) are a group of enzymes involved in the conjugation of glutathione to a wide range of xenobiotics and as such are believed to be responsible for the detoxification of a variety of substances (Jakoby, 1978, 1980). They are classified according to their iso-electric point into three groups, basic, acidic and neutral, which differ in their gene locus, amino acid sequence, and substrate specificity, and are immunologically distinct (Mannervik *et al.*, 1985; Laisney *et al.*, 1984; Jakoby, 1978). The cellular role of the GSTs is a matter of speculation; a possible role in carcinogenesis has been suggested.

They may act by converting potential carcinogens to their active forms (Seidegard *et al.*, 1986). Additionally there is evidence that they may have a role in inactivation of some carcinogens (Clapper *et al.*, 1987). GSTs have also been implicated in the development of drug resistance in some tumours by inactivating chemotherapeutic agents. Evidence for this comes from work with drug resistant cell lines (Wang & Tew, 1985; Robson *et al.*, 1987) and also studies of tumour biopsies which have suggested that those tumours which are generally resistant to chemotherapy, for example colonic carcinomas, have high levels of acidic GST, whilst those generally responsive, for example those of the head and neck, have low levels (Clapper *et al.*, 1987).

Shiratori *et al.* (1987) demonstrated increased expression of acidic GST, both nuclear and cytoplasmic, in all grades of CIN as well as invasive lesions, compared with absence of expression in normal stratified squamous epithelium. This group used a rabbit polyclonal antibody for immunohistochemical evaluation of expression and they further demonstrated enhanced v-H-*ras* gene product in many lesions showing enhanced GST expression. Shiratori proposed that GST was a potentially useful marker for pre-neoplasia of the human cervix. The purpose of the present studies was to evaluate this hyopothesis using a polyclonal antibody raised in rabbits to GST purified from a human spleen.

Correspondence: B. Angus, Department of Pathology, Royal Victoria Infirmary, Queen Victoria Road, Newcastle upon Tyne NE1 4LP, UK.
Received 5 July 1989; and in revised form 19 May 1990.

### Materials and methods

### *Cases*

Neutral buffered formalin-fixed, paraffin wax-embedded tissue was retrieved from the files of Middlesbrough General Hospital. The material studied consisted of seven normal cervices from hysterectomy specimens, 11 colposcopic biopsies showing immature squamous metaplasia, seven each of CIN1, CIN2 and CIN3 from colposcopic biopsy specimens and 28 invasive carcinomas, some of which were from cervical punch biopsies ($n = 18$) while others were from hysterectomy specimens ($n = 10$).

Routine diagnostic criteria were applied (Buckley *et al.*, 1982); briefly these are as follows: cases of immature squamous metaplasia showed a multilayered epithelium with no surface maturation, little intracytoplasmic glycogen and associated mucinous columnar epithelial cells, usually on the epithelial surface. In all cases of CIN, nuclear abnormalities characteristic of neoplasia, such as nuclear enlargement, hyperchromatism and pleomorphism, were present throughout the full thickness of the epithelium, but there was no invasion of the epithelial basement membrane. In many cases there was cytoplasmic differentiation in the upper part of the epithelium. Cases of CIN were graded according to the proportion of the epithelial thickness occupied by undifferentiated neoplastic cells of basaloid type. Thus, in CIN I undifferentiated cells occupied the lower third or less of the epithelium, in CIN II undifferentiated cells occupied between one-third and two-thirds of the epithelium, while in CIN III undifferentiated cells occupied more than two-thirds of the epithelial thickness. Cases of invasive squamous carcinoma were those in which the neoplastic squamous epithelial cells had invaded through the basement membrane into the underlying stroma. The cases of invasive carcinoma were subdivided into keratinising and non-keratinising types.

The cases of CIN were further classified according to the presence or absence of koilocytosis. Koilocytes were defined as epithelial cells showing prominent perinuclear cytoplasmic clearing, thickened cell margins and hyperchromatic nuclei with irregular outlines and uniformly distributed heterochromatin (Ludwig *et al.*, 1981).

### *Purification of glutathione S-transferase*

Acidic GST was obtained from the spleen of a patient with chronic lymphocytic leukaemia and purified using affinity chromatography and fast protein liquid chromatography (FPLC) on Mono Q. (Mannervik & Guthenberg, 1981; Habik & Jakoby, 1981). The enzyme isolated in this way has similar substrate specificities, molecular weight on SDS-PAGE and iso-electric point as the human pi form isolated from placenta (manuscript in preparation).

### *Preparation of antiserum*

Polyclonal antiserum to the GST was raised in male New Zealand white rabbits. Protein (150–200 μg) was injected into the hind quarter intramuscularly on each occasion initially with Freund's complete adjuvant. Subsequent monthly injections used Freund's incomplete adjuvant. Antiserum specificity and titre were assessed using both an ELISA technique and immunoblotting. The antiserum cross-reacts with placental and platelet GST. No cross-reactions with basic and neutral forms of GST isolated from human liver, or with other cytosolic proteins, were seen at dilutions of 1 in 1,000 (ELISA, immunoblotting) and 1 in 100 (immunocytochemistry) (manuscript in preparation).

### *Immunohistochemical staining*

The principles of the methods used have been described by Burns (1979). Three μm paraffin wax sections were cut from the tissue blocks and mounted on lysine coated slides. The sections were dried, dewaxed and rehydrated. After washing with water the sections were immersed in TRIS buffered saline for 5 min and then covered by normal swine serum diluted 1 in 5 with TRIS buffered saline. After 10 min the excess swine serum was removed and replaced by the primary anti-GST antibody at a dilution of 1 in 400. After overnight incubation at 4°C the sections were rinsed twice in TRIS buffered saline for 5 min and swine anti-rabbit secondary antibody (Dako) at a dilution of 1 in 20 applied. Following incubation for 30 min at room temperature the sections were washed in TRIS buffered saline and rabbit peroxidase-anti-peroxidase antibody (Dako) applied at 1 in 50 dilution in normal swine serum. After incubation for 30 min at room temperature the sections were rinsed in TRIS buffered saline and the peroxidase reaction developed using diaminobenzidine solution (prepared by dissolving 80 mg diaminobenzidine in 100 ml of a 0.68 g $l^{-1}$ solution of imidazole) with 1% hydrogen peroxide. Sections were incubated with this solution for between 1 and 2 min and then thoroughly washed in water, counterstained with haematoxylin and mounted. Tissue from a carcinoma of the breast was used as a positive control, while omission of primary and secondary antibodies served as negative controls.

### *Scoring*

The intensity of staining was graded as follows: (−) negative; (+) positive; (++) strongly positive. The proportion of cells showing staining was scored as follows: 1, 1–25%; 2, 26–50%, 3, 51–75%; 4, 76–100%.

The distribution of staining was assessed by scoring separately the nuclear and cytoplasmic staining. Endocervical epithelium as well as stratified squamous epithelium was examined. The basal, parabasal, intermediate and superficial layers of the stratified epithelium of the normal cervices, immature squamous metaplasia and cases of CIN were assessed separately, as were the koilocytes present in some of the cases of CIN.

For each case, all relevant tissue on the sections was examined and scored as above. Tables were constructed to allow comparison of the number of cells stained and intensity and distribution of staining in the different types of epithelium.

### Results

Koilocytosis was identified in six of the seven cases of CIN1, six of the seven cases of CIN2 and five of the seven cases of CIN3. Of the invasive carcinomas, 22 were non-keratinising while six were keratinising. Although there was some variation between individual cases, the pattern of cytoplasmic staining was similar in normal stratified squamous cervical epithelium, immature squamous metaplasia, all grades of CIN and invasive carcinomas. In most cases, cytoplasmic staining of moderate intensity (scored +) was seen in more than 50% of cells. No differences in the pattern of cytoplasmic staining between the categories studied were identified.

However, marked differences in nuclear staining between normal stratified squamous cervical epithelium, immature squamous metaplasia, CIN and invasive carcinoma were observed. Most of the normal cervices showed nuclear staining graded as + intensity of less than 25% of cells, mainly in the lower layers of the stratified squamous epithelium (Tables I and II, Figure 1). In all grades of CIN, there was more intense nuclear staining of a greater proportion of the cells (Tables I and II), and staining of nuclei in the upper epithelial layers was also noted (Figures 2–4). Immature squamous metaplasia showed a nuclear staining pattern similar to that of CIN. Nuclear staining in the invasive carcinomas was variable, but in general was less intense than in CIN, and in most cases less than 50% of the cells showed nuclear staining. No differences in nuclear staining pattern between non-keratinising and keratinising squamous carcinomas were found (Tables I and II). The koilocytes present in many of the cases of CIN showed both cytoplasmic and

**Table I** Intensity of staining of neoplastic and non-neoplastic squamous epithelium

| | *Intensity of nuclear staining* – | + | ++ | *Total* |
|---|---|---|---|---|
| Normal | 1 | 6 | 0 | 7 |
| Immature squamous metaplasia | 0 | 0 | 11 | 11 |
| CIN 1 | 0 | 1 | 6 | 7 |
| CIN 2 | 0 | 0 | 7 | 7 |
| CIN 3 | 0 | 0 | 7 | 7 |
| Invasive squamous carcinoma | | | | |
| Non-keratinising | 0 | 13 | 9 | 22 |
| Keratinising | 0 | 5 | 1 | 6 |

The numbers shown indicate the number of cases falling into each staining intensity category.

**Figure 1** Non-neoplastic cervical stratified squamous epithelium. Note weak staining in the lower one-third of the epithelium. Original magnification × 200.

**Figure 2** Stratified squamous epithelium showing CIN 1. Note intense staining of nuclei in the lower one-third of the epithelium. Original magnification × 200.

**Figure 3** Stratified squamous epithelium showing CIN II. Intense nuclear staining is seen in the upper one-third of the epithelium, with much lighter staining of the flattened surface layer and lower layers. Original magnification × 200.

a b

**Figure 4** Stratified squamous epithelium showing CIN III. Intense nuclear staining is seen in all layers, maximal in the upper two-thirds. Original magnifications: **a** × 400; **b** × 200.

**Table II** The proportion of cells showing nuclear staining in normal and neoplastic stratified squamous epithelium

| | *Proportion of cells showing nuclear staining* 0 | *1–25%* | *26–50%* | *51–75%* | *76–100%* | *Total* |
|---|---|---|---|---|---|---|
| Normal | 1 | 6 | 0 | 0 | 0 | 7 |
| Immature squamous metaplasia | 0 | 0 | 6 | 2 | 3 | 11 |
| CIN 1 | 0 | 3 | 3 | 1 | 0 | 7 |
| CIN 2 | 0 | 1 | 5 | 1 | 0 | 7 |
| CIN 3 | 0 | 0 | 4 | 3 | 0 | 7 |
| Invasive squamous ca. | | | | | | |
| Non-keratinising | 0 | 11 | 8 | 3 | 0 | 22 |
| Keratinising | 0 | 4 | 2 | 0 | 0 | 6 |

The numbers shown indicate the numbers of cases falling into each category.

nuclear staining but this did not appear to differ in intensity or distribution from that seen in the non-koilocytotic cells. Where endocervical epithelium was present, all cases showed nuclear staining (Figure 5).

### Discussion

Although we have found an association between the development of cervical neoplasia and expression of GST, our results differ from those of Shiratori *et al.* (1987), who found an increase in both cytoplasmic and nuclear expression of GST with increasing severity of neoplasia from CIN1 to invasive carcinoma. In contrast, we found no differences in cytoplasmic staining, while nuclear staining was increased in all grades of CIN, but not in invasive carcinomas. Unlike these workers, we found no association between koilocytosis and expression of GST.

The differences in these results may be due to either the specificity of the primary antibody or the technical methods used. Although both primary antibodies were shown to be specific for acidic GST, Shiratori *et al.* (1987) used antiserum raised to GST of placental origin, whereas our antiserum was raised to GST derived from the spleen of a patient with chronic lymphocytic leukaemia. Thus there may be minor differences in the specificities of the antibodies which could affect binding at some sites. Furthermore, while our tissues were fixed in formalin, most of the specimens used by Shiratori *et al.* (1987) had been fixed in 90% ethanol, although a few of their cases were fixed in 10% formalin: 90% ethanol. Thus, differences in fixation may also account for differences in the immunohistochemical staining patterns. Indeed, Shiratori *et al.* reported that nuclear staining was stronger in ethanol-fixed tissues than in formalin: ethanol fixed tissues and it is possible therefore that fixation may also affect cytoplasmic staining.

Finally, in this study a peroxidase–antiperoxidase method was used, while Shiratori *et al.* used avidin–biotin–peroxidase.

The role of GST in neoplastic transformation is not clear. Our findings do not suggest that it is related to cell proliferation, since expression was less in the invasive carcinomas than in the cases of CIN. Furthermore, GST expression was often maximal in the upper epithelial layers in cases of CIN, including CIN1, whereas proliferation would be expected to be greatest in the lower layers.

GST may be involved in the process of carcinogenesis, perhaps by carcinogen activation or inactivation (Wang & Tew, 1985; Robson *et al.*, 1987; Seidegard *et al.*, 1986) or in response to viral infection. This function may become obsolete once malignant transformation has occurred, explaining the decreased nuclear expression which we found in invasive carcinomas.

Our studies indicate that GST would be of limited use as a marker of cervical neoplasia in cervical cytology screening by either conventional or automated methods. The increased nuclear expression of GST was consistent, in that the intensity and proportion of cells showing nuclear staining was increased in all but one case of CIN. However, endocervical cells and immature squamous metaplasia also express GST, and these would have to be distinguished from neoplastic squamous cells. It is usually possible to identify endocervical cells visually in a cervical smear preparation, but an automated process would require a second marker. For example, nuclear GST positive cells could be evaluated for DNA content (Millet *et al.*, 1982), or endocervical cells discounted by staining for an antibody specific for simple epithelium. The expression of GST by immature squamous metaplasia is particularly disappointing as this lesion may be mistaken for CIN on both cytology and histology.

**Figure 5** Endocervical glands. Note intense nuclear staining. Original magnification × 200.

A further potential disadvantage of GST as a marker of transformation is that it would possibly not detect invasive carcinomas. This is not certain, however, as we have not yet evaluated GST expression in exfoliated cells. Furthermore, Shiratori *et al.* (1987) demonstrated enhanced expression in invasive carcinoma as well as pre-invasive lesions.

Although GST may be of use in diagnostic cytology, it seems likely that more specific markers of transformation, such as oncogene or tumour suppressor gene nuclei acid or protein (Spandidos & Anderson, 1989) may prove to be more valuable in this area. As well as immunohistochemistry, techniques such as *in situ* hybridisation, applied successfully to detection of viral nucleic acid in cervical lesions (Burns *et al.*, 1987) have potential use.

In conclusion, GST shows lack of specificity for CIN and possibly also lack of sensitivity for invasive carcinoma and therefore is of little use as a marker of transformation in the human cervix uteri.

We are grateful to Mrs Jacqueline Richards for technical assistance and to Mrs Elizabeth Tweedy for secretarial assistance. We would also like to thank Mr John Cairns for his advice on the immunohistochemical aspects of the study. Dr Alex Cattan is supported by the Tyneside Leukaemia Research Fund and Dr Andy Hall is supported by the Leukaemia Research Fund.

### References

ANGUS, B., KIBERU, S., PURVIS, J., WILKINSON, L. & HORNE, C.H.W. (1988). Cytokeratins in cervical dysplasia and neoplasia: a comparative study of immunohistochemical staining using monoclonal antibodies NCL-5D3, CAM 5.2 and PKK1. *J. Pathol.*, **155,** 71.

ANGUS, B., PURVIS, J., STOCK, D. & 5 others (1987). NCL-5D3: a new monoclonal antibody recognizing low molecular weight cytokeratin effective for immunohistochemistry using fixed paraffin-embedded tissue. *J. Pathol.*, **153,** 377.

BOBROW, L.G., MAKIN, C.A., LAW, S. & BODMER, W.F. (1986). Expression of low molecular weight cytokeratin proteins in cervical neoplasia. *J. Pathol.*, **B148,** 135.

BRESCIA, R.J., JENSON, A.B., LANCASTER, W.D. & KURMAN, R.J. (1986). The role of human papillomaviruses in the pathogenesis and histologic classification of precancerous lesions of the cervix. *Human Pathol.*, **17,** 552.

BUCKLEY, C.H., BUTLER, E.B. & FOX, H. (1982). Cervical intra-epithelial neoplasia. *J. Clin. Pathol.*, **35,** 1.

BURNS, J. (1979). Immunohistochemical methods and their application in the routine laboratory. In *Recent Advances in Histopathology 10*, Anthony, P.P. & MacSween, R.N.W. (eds) p. 337. Churchill Livingstone: Edinburgh, London, Melbourne & New York.

BURNS, J., GRAHAM, A.K., FRANK, C., FLEMING, K.A., EVANS, M.F. & MCGEE, J.O.D. (1987). Detection of low copy human papillomavirus DNA and RNA in routine paraffins sections of cervix by non-isotopic in-situ hybridisation. *J. Clin. Pathol.*, **40,** 858.

CLAPPER, M.L., BULLER, A.L., SMITH, T.M. & TEW, K.T. (1987). Glutathione S-transferases in alkylating agent resistant cells. In *Glutathione S-transferases and Carcinogenesis*, Mantle, T.J., Pickett, C.B. & Hayes, J.D. (eds) p. 213. Taylor and Francis: London.

EGAN, M., FREETH, M. & CROCKER, J. (1988). Intraepithelial neoplasia, human papillomavirus infection and argyrophilic nucleoprotein in cervical epithelium. *Histopathology*, **13**, 561.

ELLIOTT, P.M., TATTERSALL, M.H.N., COPPLESON, M. & 7 others (1989). Changing character of cervical cancer in young women. *Br. Med. J.*., **298**, 288.

FRAY, R.E., HUSAIN, O.A.N., TO, A.C.W. & 5 others (1984). The value of immunohistochemical markers in the diagnosis of cervical neoplasia. *Br. J. Obstet. Gynaecol.*, **91**, 1037.

HABIK, W.H. & JAKOBY, W.B. (1981). Assays for differentiation of glutathione-S-transferase. *Meth. Enzymol.*, **77**, 398.

JAKOBY, W.B. (1978). The glutathione S-transferases: a group of multifunctional detoxification proteins. *Adv. Enzymol.*, **46**, 383.

JAKOBY, W.B. (ed.) (1980). *Enzyme Basic of Detoxification, Vols 1 and 2*. Academic Press: New York.

LAISNEY, V., CONG, N.V., GROSS, M.S. & FREZAL, J. (1984). Human genes for glutathione S-transferases. *Hum. Genet.*, **68**, 221.

LUDWIG, M.E., LOWELL, D.M. & LIVOLSI, V.A. (1981). Cervical condylomatous atypia and its relationship to cervical neoplasia. *Am. J. Clin. Pathol.*, **76**, 255.

MCCANCE, D.J. (1986). Human papillomavirus and cancer. *Biochim. Biophys. Acta*, **283**, 205.

MANNERVIK, B., ALIN, P., GUTHENBERG, C. & 4 others (1985). Identification of three classes of cytosolic glutathione transferase common to several mammalian species: correlation between structural data and enzymatic properties. *Proc. Natl Acad. Sci USA*, **82**, 7702.

MANNERVIK, B. & GUTHENBERG, C. (1981). Glutathione transferase (human placenta). *Meth. Enzymol.*, **77**, 231.

MILLET, J.A., HUSAIN, O.A.N., BITENSKY, L. & CHAYEN, J. (1982). Feulgen hydrolysis particles in cells exfoliated from the cervix uteri: a potential aid in the diagnosis of malignancy. *J. Clin. Pathol.*, **35**, 345.

MORRIS, H.B., GATTER, K.C., PULFORD, K. & 5 others (1983). Cervical wart virus infection, intraepithelial neoplasia and carcinoma; an immunohistological study using a panel of monoclonal antibodies. *Br. J. Obstet. Gynaecol.*, **90**, 1069.

RAJU, G.C. (1988). Expression of the cytokeratin marker CAM 5.2 in cervical neoplasia. *Histopathology*, **12**, 437.

ROBSON, C.N., LEWIS, A.D., WOLF, C.R. & 5 others (1987). Reduced levels of drug-induced DNA cross-linking in nitrogen mustard-resistant chinese hamster ovary cells expressing elevated glutathione S-transferase activity. *Cancer Res.*, **47**, 6022.

SEIDEGARD, J., PERO, R.W., MILLER, D.G. & BEATTIE, E.J. (1986). A glutathione transferase in human leukocytes as a marker for the susceptibility to lung cancer. *Carcinogen*, **7**, 751.

SHIRATORI, Y., SOMA, Y., MARUYAMA, H., SATO, S., TAKONO, A. & SATO, K. (1987). Immunohistochemical detection of the placental form of glutathione S-transferase in dysplastic and neoplastic human uterine cervix lesions. *Cancer Res.*, **47**, 6806.

SINCOCK, A.M., MIDDLETON, J. & MONCRIEFF, D. (1983). Towards an automated procedure for the quantitative cytological screening of cervical neoplasms. *J. Clin. Pathol.*, **36**, 535.

SPANDIDOS, D.A. & ANDERSON, M.L. (1989). Oncogenes and oncosuppressor genes: their involvement in cancer. *J. Pathol.*, **157**, 1.

SPRIGGS, A.L. & BODDINGTON, M.M. (1980). Progression and regression of cervical lesions. *J. Clin. Pathol.*, **33**, 517.

STERRETT, G.F., ALESSANDRI, L.M., PIXLEY, E. & KULSKI, J.K. (1987). Assessment of precancerous lesions of the uterine cervix for evidence of human papillomavirus infection: a histological and immunohistochemical study. *Pathology*, **19**, 84.

WANG, A.L. & TEW, K.D. (1985). Increased glutathione S-transferase activity in a cell line with acquired resistance to nitrogen mustards. *Cancer Treat Rep.*, **69**, 677.

WHITTAKER, J.R., SAMY, A.M.J., SUNTER, J., SINHA, D.P. & MONAGHAN, J.M. (1989). Cytokeratin expression in cervical epithelium: an immunohistological study of normal wart virus infected and neoplastic tissue. *Histopathology*, **14**, 151.

YOUNG, L.S., BEVAN, I.S., JOHNSON, M.A. & 4 others (1989). The polymerase chain reaction: a new epidemiological tool for investigating cervical human papillomavirus infection. *Br. Med. J.*, **298**, 14.